# Statins in adult patients with HIV: Protocol for a systematic review and network meta-analysis

**DOI:** 10.1097/MD.0000000000010116

**Published:** 2018-04-13

**Authors:** Leonardo Roever, Elmiro Santos Resende, Angélica lemos Debs Diniz, Nilson Penha-Silva, João Lucas O’Connell, Paulo Fernando Silva Gomes, Hugo Ribeiro Zanetti, Anaisa Silva Roerver-Borges, Fernando César Veloso, Thiago Montes Fidale, Antonio Casella-Filho, Paulo Magno Martins Dourado, Antonio Carlos Palandri Chagas, Sadeq Ali-Hasan-Al-Saegh, Paulo Eduardo Ocke Reis, Rogério de Melo Pinto, Gustavo B. F. Oliveira, Álvaro Avezum, Mansueto Neto, André Durães, Rose Mary Ferreira Lisboa da Silva, Antonio José Grande, Celise Denardi, Renato Delascio Lopes, Nitesh Nerlekar, Shahab Alizadeh, Adrian V. Hernandez, Giuseppe Biondi-Zoccai

**Affiliations:** a Federal University of Uberlândia, Department of Clinical Research, Heart Institute (InCor), Master Institute of Education President Antonio Carlos, IMEPAC, Araguari; b Department of Clinical Research, HCFMUSP—University of São Paulo Medical School, Department of Cardiology, São Paulo, Brazil; c and Faculty of Medicine ABC, Department of Cardiology Santo André; d Cardiovascular Research Center, Shahid Sadoughi University of Medical Sciences, Department of Cardiology, Yazd, Iran; e Department of Specialized and General Surgery, Fluminense Federal University, Rio de Janeiro, Brazil; f Dante Pazzanese Institute of Cardiology; g Dante Pazzanese Institute of Cardiology, Department of Clinical Research São Paulo, Brazil; h Graduate Program in Medicine and Health, Department of Heath and Sciences, Federal University of Bahia; i Federal University of Minas Gerais, Department of Cardiology, MG; j Federal University of Mato Grosso, MT, Department of Medicine. Brazil; k FOP Unicamp, Department of Clinical Research; Division of Cardiology, Duke University Medical Center, Department of Clinical Research, Durham, NC; l Monash Cardiovascular Research Centre and MonashHeart, Department of Cardiology, Clayton, Victoria, Australia; m Tehran University of Medical Sciences, Department of Medicine, University of Connecticut/Hartford Hospital Evidence-Based Practice Center, Hartford; n Department of Comparative Effectiveness and Outcomes Research Health Outcomes, CT; o Department of Medico-Surgical Sciences and Biotechnologies, Sapienza University of Rome, Latina; p Department of AngioCardioNeurology, IRCCS Neuromed, Pozzilli, Italy.

**Keywords:** HIV, network meta-analysis, statins

## Abstract

**Background:**

Patients with HIV have been found to suffer from lipid abnormalities, including elevated levels of total and LDL-cholesterol as well as triglyceride levels. Abnormal lipid levels are associated with an increased risk of developing cardiovascular diseases, which are significant causes of mortality among the general population. Therefore, the objective of the current study is to conduct a systematic review with network meta-analysis to compare the effects of statins classes on HIV patients.

**Methods:**

Randomized clinical trials (RCTs) and observational studies published in English up to 31 December 2017, and which include direct and/or indirect evidence, will be included. Studies will be retrieved by searching four electronic databases and cross-referencing. Dual selection and abstraction of data will occur. The primary outcome will all-cause mortality, new event of acute myocardial infarction, stroke (hemorrhagic and ischemic), hospitalization for acute coronary syndrome and urgent revascularization procedures and cardiovascular mortality. Secondary outcomes will be assessment of the differences in change of total cholesterol (TC), low-density lipoprotein (LDL-C), apolipoprotein B (ApoB), high density lipoprotein (HDL-C). Risk of bias will be assessed using the Cochrane Risk of Bias assessment instrument for RCTs and the Strengthening the Reporting of Observational Studies in Epidemiology instrument for observational studies. Network meta-analysis will be performed using multivariate random-effects meta-regression models. The surface under the cumulative ranking curve will be used to provide a hierarchy of statins that reduce cardiovascular mortality in HIV patients. A revised version of the Cochrane Risk of Bias tool (RoB 2.0) will be used to assess the risk of bias in eligible RCTs. Results will be synthesized and analyzed using network meta-analysis (NMA). Overall strength of the evidence and publication bias will be evaluated. Subgroup and sensitivity analysis will also be performed.

**Results and Conclusion:**

Ethics approval was not required for this study because it was based on published studies. The results and findings of this study will be submitted and published in a scientific peer-reviewed journal. The evidence will determine which combination of interventions are most promising for current practice and further investigation.

**Trial registration number:**

PROSPERO (CRD42017072996).

## 1 Introduction

### 1.1 Rationale

With the advent of antiretroviral therapy (ART) human immunodeficiency virus (HIV)-infected patients are experiencing a significant increase in life expectancy. However, as this population is aging, it becomes increasingly clear to morbidity and mortality caused by events unrelated to HIV infection and/or treatment. One of the possible explanations of this characteristic is associated to the effects of the ART on the lipid metabolism, with increase of LDL-C, triglycerides, and total cholesterol.^[1]^ In addition to the ART cholesterol side effects, HIV per si provides reduction in HDL-C.^[2]^

In addition, the incidence of sudden cardiac death in HIV-infected patients is significantly higher when compared to the general population with similar risk factors.^[2]^

Consequently, a need exists for a meta-analysis that includes RCTs studies so that adverse outcomes can be appropriately documented. In addition, it has recently been suggested that RCTs and observational studies should not be considered in isolation. Furthermore, additional studies may have been published since the previous studies search for eligible trials (December 31, 2017).

### 1.2 Objective

The primary objective of this study is to conduct a systematic review with network meta-analysis of randomized trials to compare the effects of different pharmacological classes of statins on cardiovascular (CV) mortality. The network meta-analytic approach is appropriate here because it allows for the inclusion of multiple interventions from both direct and indirect comparisons that have not been examined in a head-to-head fashion.

## 2 Methods

### 2.1 Overview

This study will follow the Preferred Reporting Items for Systematic Reviews and Meta-Analyses guidelines^[3]^ for meta-analyses of healthcare interventions and the current protocol report follows the Preferred Reporting Items for Systematic Reviews and Meta-Analyses Protocols.^[4]^ This protocol is registered in International Prospective Register of Systematic Reviews (trial registration number: CRD42017072996).

### 2.2 Eligibility criteria

Studies that meet the following criteria will be included: randomized trials and observational studies; adults ≥18 years of age with HIV, either with or without a history of CV disease: at least 1 oral statins intervention group; data on CV mortality and/or major adverse cardiac events; studies published in English up to December 31, 2017. The decision to include patients with HIV with or without a history of CV disease was made based on our preliminary search of clinical trials that included patients with either a history of CV disease or those who are at a heightened risk for CV disease. Major adverse cardiac events will be defined as an incidence of acute myocardial infarction (AMI), stroke, hospitalization for acute coronary syndrome, and urgent revascularization procedures.

### 2.3 Information sources

The following databases will be searched from their inception forward for potentially eligible studies in English language published on or before January 31, 2017: PubMed, Scopus, Web of Science, Cochrane Central Register of Controlled Clinical Trials, clinical trials registry (ClinicalTrials.gov). In addition, cross-referencing from retrieved studies will be conducted.

### 2.4 Search strategy

Search strategies adapted from a previous research^[3]^ will be developed using text words and Medical Subject Headings. Electronic databases will be searched for studies on the effects of statins on CV safety in adults with HIV. The first author will conduct all database searches. The search strategy for all other databases will be adapted based on the requirements of each database.

### 2.5 Study selection

**Figure 1:**
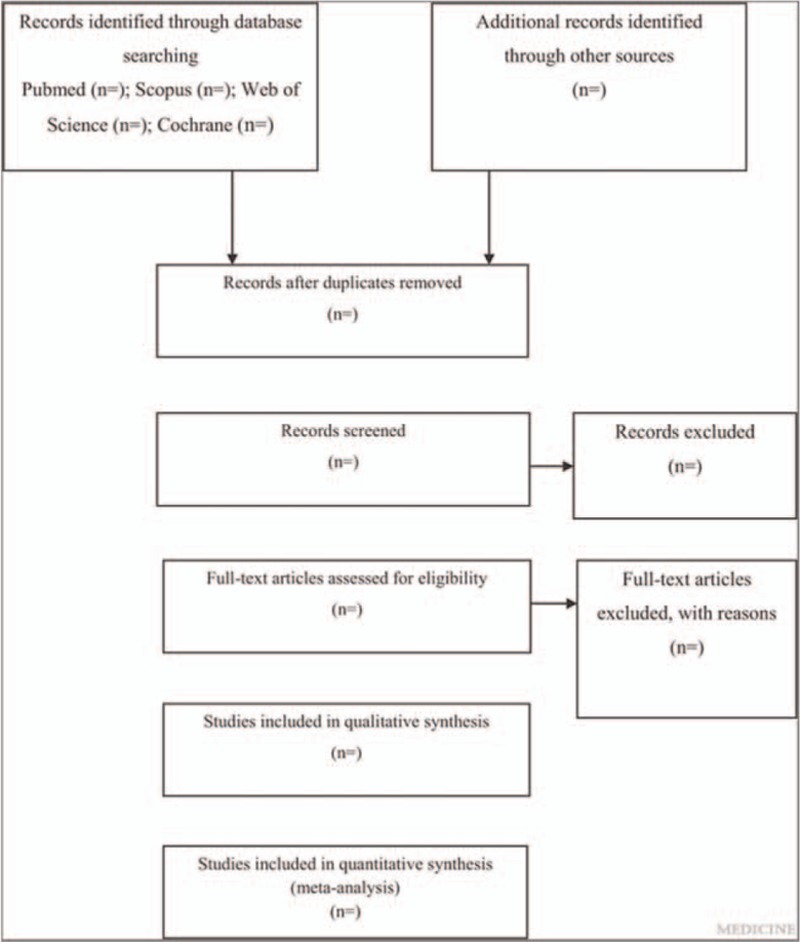
Flow diagram of study selection process.

All studies extracted from electronic databases using the search strategy will be imported into EndNote V.X7.5. Duplicate studies will be removed electronically using the “Find Duplicates” tool in EndNote. The studies will be examined again manually to find and delete any additional duplicates. The first 2 authors will select studies independent of each other. Complete articles will be obtained for all titles and abstracts that appear to meet the inclusion criteria or where there is any uncertainty. Reasons for exclusion will be coded as one or more of the following: inappropriate population, inappropriate intervention, inappropriate comparison, inappropriate outcome(s), inappropriate study design, and other. After selection, the first 2 authors will review their selections and resolve any discrepancies by consensus. If consensus cannot be reached, the third author will be consulted. The overall agreement rate prior to correcting discrepant items will be calculated using Cohen's kappa (κ) statistics. Once discrepancies are resolved, the overall precision of searches will be calculated by dividing the number of studies included by the total number of studies screened after removing duplicates. The number needed to read will then be calculated as the inverse of the precision. A flow diagram that depicts the search process and an online supplementary file that includes a reference list of all studies excluded (including the reason(s) for exclusion) will be included in the study. The proposed structure for the flow diagram is shown in Figure 1.

### 2.6 Data abstraction

Before initiating data abstraction, a codebook will be developed in Microsoft Excel 2013. The codebook will be developed by the first author with input from the third author. The major categories of variables to be coded will include: study characteristics (author, journal, year, etc.); participant characteristics (age, sex, CV disease at baseline, etc.); intervention characteristics (pharmacological class of statins, dose, etc.); control characteristics; outcome data for CV mortality, all-cause mortality, incidence of AMI, stroke, hospitalization for acute coronary syndrome, and urgent revascularization procedures. The first 2 authors will abstract data from selected studies, independent of each other, using the codebook in Microsoft Excel. On completion, both authors will review the codebooks and resolve discrepancies by consensus. If consensus cannot be reached, the third author will provide a recommendation. Prior to correcting disagreements, the overall agreement rate will be calculated using Cohen's κ statistic.

### 2.7 Outcomes and prioritization

Risk of bias for RCTs will be assessed using the Cochrane Risk of Bias instrument.^[5,6]^ Bias in RCTs will be evaluated for 6 domains: random sequence generation, allocation concealment, blinding of participants and personnel, blinding of outcome assessors, incomplete outcome data, and selective reporting. Each study will be classified as having a high, low, or unclear risk of bias overall and for each domain. The overall risk of bias will be classified as high if any one of the domains is considered high risk. The first 2 authors will conduct all risk of bias assessments independent of each other. The 2 authors will then review the results for risk of bias assessment and resolve any discrepancies by consensus. If consensus cannot be reached, the third author will be consulted.

### 2.8 Risk of bias assessment

We will choose the Cochrane Collaboration's risk of bias tool to evaluate the methodological quality of RCTs. The risk of bias tool consists of 6 domains: sequence generation, allocation concealment, blinding, incomplete data, selective reporting, and other bias. Two independent reviewers (LR and HRZ) will independently evaluate the quality of RCTs. Sequence generation will be considered as adequate if central randomization or tables of random numbers are used. Allocation concealment will be considered as adequate if central randomization or sealed envelopes are used. We will consider blinding as adequate if participants, outcome assessors, and statisticians are blinded from the group assignment. The other domains will be assessed exactly as the criteria of the risk of bias tool. A summary of risk of bias of all the 6 domains will be provided for each trial. We choose to consider sequence generation, allocation concealment, and blinding as the key essential domains to score the overall quality of a trial. Discrepancies among the 2 reviewers (LR and HRZ) will be solved by discussion or will be judged by a third reviewer (GBZ).

## 3 Data synthesis

### 3.1 Calculation of effect sizes

All analyses will be conducted using the natural log of OR and then transformed back to ORs for presentation purposes. If OR is not reported, it will be calculated from data reported in the study. If data are not available to calculate OR, it will be requested from the study authors. Secondary outcomes will be calculated using the same procedure as for our primary outcome. If a study includes both direct and indirect comparisons, only direct comparison data will be included given that the primary focus of the present study is to compare the CV safety between different statins. The data augmentation approach will be used to make direct comparisons if the control group is placebo.^[7]^ In this technique, direct evidence studies that lack a control (placebo) group will have one generated from the weighted average of the arm-specific means and SD.^[8]^

### 3.2 Pooled estimates for change in outcomes

Network maps will be drawn to depict the treatments that are directly compared against each other and the amount of evidence available for each treatment and its comparator. Separate network maps will be presented for each outcome. Contribution plots for each outcome will be generated to determine the most dominant comparisons for each network estimate, as well as for the entire network. The weights applied will be a function of the variance of the direct treatment effect and the network structure, the product being a per cent contribution of each direct comparison to each network estimate. Network and contribution plots will be produced using the network plot and net weight commands, respectively,^[9]^ in Stata/IC for Mac V.14.0 (STATA; 2016).

Prior to conducting network meta-analysis, pairwise meta-analysis using random-effects models will be conducted in order to examine statistical heterogeneity within each comparison.^[10]^ Heterogeneity will be assessed using Cochran's *Q* statistics and *I*^2^, an extension of *Q*.^[11,12]^ A *Q* statistic ≤0.10 and/or an *I*^2^ value >50% will be considered to represent significant heterogeneity. On completion of pairwise meta-analysis, network meta-analysis will be performed using multivariate random-effects models based on the mvmeta command in Stata/IC for Mac V.14.0.^[12]^ Nonoverlapping 95% CIs will be considered to represent statistically significant changes. Separate network meta-analysis models will be used to compare CV mortality, all-cause mortality, incidence of AMI, stroke, hospitalization for acute coronary syndrome, and urgent revascularization procedures.

Subgroup analyses will be conducted to examine the association between our primary outcome and oral statins. These will include year of drug approval by the US FDA, the presence or absence of CV disease risk at baseline, lipids at the baseline, number of comorbidities, type of treatment (monotherapy, dual therapy, or triple therapy) and the country the study was conducted in. Secondary outcomes will be handled using the same approach.

We will examine the consistency of the estimates of treatment effects from direct and indirect evidence for each outcome using the mvmeta command in Stata.^[8]^ An alpha value ≤0.05 will be considered to represent statistically significant inconsistency. Prediction intervals will be used to enhance the interpretation of findings and provide an estimate of expected results in a future study.^[8–12]^ Prediction intervals will be generated using the mvmeta and interval plot^[9]^ commands in Stata/IC for Mac V.14.0.

### 3.3 Meta biases

Small-study effects (publication bias, etc.) will be assessed using comparison-adjusted funnel plots. Unlike traditional funnel plots in pairwise meta-analysis, funnel plots in network meta-analysis need to account for the fact that studies estimate treatment effects for different comparisons. Consequently, there is no single reference line from which symmetry can be evaluated. For the comparison-adjusted funnel plot, the horizontal axis will represent the difference between study-specific effect sizes from the comparison-specific summary effect. In the absence of small-study effects, the comparison-adjusted funnel plot should be symmetric around the zero line. Since the treatments need to be organised in some meaningful way to examine how small studies may differ from large ones, comparisons will be defined so that all refer to an active treatment versus a control group. Comparison-adjusted funnel plots will be generated using the netfunnel command^[9]^ in Stata/IC for Mac V.14.0.

Transitivity (similarity in the distribution of potential effect modifiers across the different pairwise comparisons)^[13]^ will be evaluated using random-effects network meta-regression while controlling for the different study designs within each comparison. Potential effect modifiers will include age, gender, baseline lipids, duration of HIV, obesity, the presence of CV disease at baseline, and medication status. In addition, because individuals taking medication are more likely to have severe disease or more comorbidity than those without medication, we will also include baseline condition of the patient (e.g., disease severity) in our regression models. However, since this is an aggregate data meta-analysis and if the patients included within each study are heterogeneous (e.g., different levels of disease severity within the same study), we will include as a covariate those studies that control for such factors versus those that do not. Table 1 provides a complete list of covariates that we plan to include. Transitivity analysis will be conducted using the mvmeta command^[8]^ in Stata/IC for Mac V.14.0.

**Table 1:**
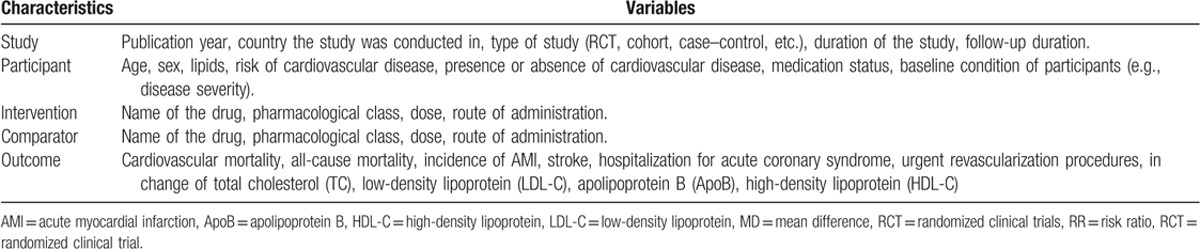
Covariates that will be included in the study.

Ranking analysis is a major advantage of network meta-analysis because it allows one to rank all interventions for the outcome of interest. For the current study, we will generate ranking plots for a single outcome using probabilities.^[14,15]^ However, since ranking treatments based solely on the probability of each treatment being the best does not account for the uncertainty in the relative treatment effects and the potential for assigning higher ranks in which little information is available, rankograms and cumulative ranking probability plots will be used to show ranking probabilities along with their uncertainty for changes in our primary and secondary outcomes.^[14,15]^ Surface under the cumulative ranking curves (SUCRA), a transformation of mean ranks, will be used to provide a hierarchy of treatments while accounting for the location and variance of all treatment effects.^[14,15]^ Larger SUCRA values are indicative of better ranks for the treatment. Separate ranking analyses will be conducted for all primary and secondary outcomes using the mvmeta^[8]^ and SUCRA^[9]^ commands in Stata/IC for Mac V.14.0.

### 3.4 Software used for data synthesis

All data will be analyzed using Stata/IC for Mac V.14.0.

### 3.5 Confidence in the cumulative evidence

Strength in the body of evidence will be assessed using the Grading of Recommendations Assessment, Development and Evaluation (GRADE) instrument for network meta-analysis.^[16]^ Two main outputs are reported in a network meta-analysis: pairwise effect estimates and treatment rankings. Since the 2 outputs are generated using different techniques, they may differ between each other. Therefore, it is important to assess the level of confidence to be placed on each output. The level of confidence will be assessed using GRADE across 4 domains: study limitations, joint consideration of indirectness and transitivity, joint consideration of statistical heterogeneity and statistical inconsistency, and imprecision and publication bias. Based on these assessments, the overall strength of evidence will be ranked as either high, moderate, low, or very low. The overall confidence will be classified as high if any one of the domains is considered high.

### 3.6 Statistical analysis

The data for statistical analysis will be extracted into an Excel file. The primary outcome is continuous data, so we will calculate the effect size of the interventions using the standardized mean difference (SMD). For trials that present mean values of each time point, we will use the primary outcome adjusted by the baseline values. If the trials present the value of the primary outcome changing from baseline, we will calculate the SMD directly. We will calculate the 95% CI for each single SMD, and the results will be pooled using the random-effect model. The proportion of responders represents dichotomous data, so we will calculate the effect size using the relative ratio (RR). The RR and the 95% CI of each intervention will be calculated and pooled using the random-effect model.

The network meta-analysis will be conducted using the “netmeta” package in the R software (http://www.r-project.org/), to combine direct and indirect evidence of interventions for migraine prophylaxis.^[6]^ The package is developed on the basis of the frequentist method, using the graph-theoretical method developed according to the electrical network theory.^[7]^ The first advantage of this method is that it can combine direct and indirect evidence in trials with more than 2 study arms. Multiarm studies are often included in a network meta-analysis. In these studies, the treatment effects on different comparisons are correlated, which is not fully addressed by the generalized linear mixed models^[8]^ or the Bayesian Markov Chain Monte Carlo method^[9,10]^ which is commonly used for network meta-analysis. The “netmeta” package accounts for the correlated treatment effects by reweighting all comparisons of each multiarm study. The second advantage of this method is that it provides solutions for testing the consistency of the network using Cochrane's *Q* statistics and finding out the reasons for the consistency by a net-heat plot. So we will use this method to address the consistency of the network. If the data are not suitable to carry out the synthesis, we will perform a descriptive review and summarize the evidence. The evidence strength will be assessed using the GRADE method generated by the Cochrane library. A funnel plot will be drawn to detect if there is any publication bias.

### 3.7 Dealing with missing data

There will be missing data in the trials that we included. We will first contact the authors to ask for original data by email or phone calls, if possible. If the original data are not available, we will try to calculate the data through the available coefficients; for example, we will calculate the SD from the 95% CI, *P* or *t* values. Imputations of the data will be tested in the following sensitivity analysis.

### 3.8 Subgroup analysis

To address the potential heterogeneity and inconsistency across trials, we will perform a subgroup analysis. This include subtypes of dyslipidemia (isolated hypercholesterolemia, isolated hypertriglyceridemia, mixed hyperlipidemia, and isolated HDL-cholesterol reduction), blinding method (open trial, single blind for participants, double blind for both participants and care providers), quality of evidence (high risk, unclear of the risk and low risk), duration of HIV, and mean age of the participants. Meta-regression models will be used to quantify the difference between subgroups and test for statistical significance.

### 3.9 Sensitivity analysis

Sensitivity analysis will be performed to first address whether the combined estimates of the interventions are dominated by 1 or several trials, especially those with a high risk of bias. Then we will exclude the trials to test the robustness of our study result. Second, we will test whether the imputation of the missing values affects the result of the meta-analysis. We will also test different coefficients that are used to impute the missing value; if both SE and 95% CI are available to calculate SD, we will test which is better.

### 3.10 Assessment of heterogeneity

Heterogeneity, which plays a pivotal role in both standard meta-analyses and network meta-analyses, refers to the degree of disagreement between study specific treatment effects and constitutes the basis of inconsistency. To test the heterogeneity of each pairwise comparison, we will use the *I*^2^ statistic.

### 3.11 Assessment of transitivity and similarity

In addition to the heterogeneity assessment using the *I*^2^ statistic, the assumption of transitivity and similarity based on clinical and methodological characteristics will be assessed. It should be noted that it is difficult to identify these effect modifiers using statistical analysis. We will assume that intervention effects are transitive in this network meta-analysis because we will only focus on antiepileptic drugs, and we will investigate similarity based on clinical characteristics, such as antiepileptic drug dose, period of treatment, and severity of pain symptoms at baseline, as well as according to methodological characteristics, such as study quality.^[17]^ All of these effect modifiers will be judged and reported before the network meta-analysis is conducted.

### 3.12 Assessment of inconsistency

Evaluation and explanation of inconsistency is another basic objective of a network meta-analysis. In this context, inconsistency refers to the degree of difference between direct and indirect comparisons and can be evaluated only when a loop exists in the evidence network. This means that inconsistency assessment using a design by treatment interaction model cannot be conducted if the structure of this network is a “star network” (i.e., all interventions have a single mutual comparator, such as a placebo).^[18]^ For such cases, we will test inconsistency using a node splitting model.^[19]^

To identify inconsistency among the included trials of the network, we will use Stata, performing the *Z* test to compare direct and indirect summary effects in specific loops.^[20]^ If there is no inconsistency between loops or designs, we will use a consistency model to calculate the data. For cases of significant incoherence, we will initially look for data extraction errors in loops that present inconsistency and in comparisons with large heterogeneity.^[21]^ After the data have been scrutinized, we will investigate possible sources of inconsistency within the clinical and methodological variables suspected of being potential sources of either heterogeneity or incoherence in each comparison specific group of trials. If an important inconsistency cannot be explained, we will consider avoiding synthesis of the related network.

### 3.13 Additional analyses

To ensure the quality of this review, studies not reporting blinding will be excluded prior to data synthesis because blinding plays a vital important role in the RCT. We will assess heterogeneity quantitatively using the *I*^2^ statistic, and if an *I*^2^ value is >50%, we will explore the source of heterogeneity. We will initially perform sensitivity analysis by excluding trials rated as having a high risk of bias. Additionally, meta-regression or subgroup analysis will be used to explore possible sources of heterogeneity if the number of included trials is sufficient. For network meta-regression, we will use a random effects network meta-regression model to examine potential factors.

## 4 Discussion

This network meta-analysis is expected to provide a ranking of the interventions from guideline recommendations for migraine prophylaxis, based on comparative effectiveness evidence. We also hope that the result would be of interest to the policymakers of health insurance; this might help them to make a better choice of the interventions that should be covered by insurance. Therefore, this evidence will help patients and clinicians to make decisions in such settings. The results will also aid to the development and optimization of new interventions.

## Author contributions

**Conceptualization:** Leonardo Roever, Elmiro Santos Resende, Angélica Lemos Debs Diniz, Nilson Penha-Silva, João Lucas O’Connell, Paulo Fernando Silva Gomes, Hugo Ribeiro Zanetti, Anaisa Silva Roever-Borges, Fernando César Veloso, Thiago Montes Fidale, Antonio Casella-Filho, Paulo Magno Martins Dourado, Antonio Carlos Palandri Chagas, Sadeq Ali-Hasan-Al-Saegh, Paulo Eduardo Ocke Reis, Rogério de Melo Costa Pinto, Gustavo B. F. Oliveira, Álvaro Avezum, Mansueto Neto, André Durães, Rose Mary Ferreira Lisboa da Silva, Antonio José Grande, Celise Denardi, Renato Delascio Lopes, Nitesh Nerlekar, Shahab Alizadeh, Adrian V. Hernadez, Giuseppe Biondi-Zoccai.

**Formal analysis:** Leonardo Roever.

**Methodology:** Leonardo Roever, Angélica Lemos Debs Diniz, Anaisa Silva Roever-Borges, Thiago Montes Fidale, Rogério de Melo Costa Pinto, Antonio José Grande, Nitesh Nerlekar.

**Validation:** Leonardo Roever.

**Writing – original draft:** Leonardo Roever, Elmiro Santos Resende, Angélica Lemos Debs Diniz, Nilson Penha-Silva, João Lucas O’Connell, Paulo Fernando Silva Gomes, Hugo Ribeiro Zanetti, Anaisa Silva Roever-Borges, Fernando César Veloso, Thiago Montes Fidale, Antonio Casella-Filho, Paulo Magno Martins Dourado, Antonio Carlos Palandri Chagas, Sadeq Ali-Hasan-Al-Saegh, Paulo Eduardo Ocke Reis, Rogério de Melo Costa Pinto, Gustavo B. F. Oliveira, Álvaro Avezum, Mansueto Neto, André Durães, Rose Mary Ferreira Lisboa da Silva, Antonio José Grande, Celise Denardi, Renato Delascio Lopes, Nitesh Nerlekar, Shahab Alizadeh, Adrian V. Hernadez, Giuseppe Biondi-Zoccai.

**Writing – review & editing:** Leonardo Roever, Elmiro Santos Resende, Angélica Lemos Debs Diniz, Nilson Penha-Silva, João Lucas O’Connell, Paulo Fernando Silva Gomes, Hugo Ribeiro Zanetti, Anaisa Silva Roever-Borges, Fernando César Veloso, Thiago Montes Fidale, Antonio Casella-Filho, Paulo Magno Martins Dourado, Antonio Carlos Palandri Chagas, Sadeq Ali-Hasan-Al-Saegh, Paulo Eduardo Ocke Reis, Rogério de Melo Costa Pinto, Gustavo B. F. Oliveira, Álvaro Avezum, Mansueto Neto, André Durães, Rose Mary Ferreira Lisboa da Silva, Antonio José Grande, Celise Denardi, Renato Delascio Lopes, Nitesh Nerlekar, Shahab Alizadeh, Adrian V. Hernadez, Giuseppe Biondi-Zoccai.
